# mmu_circ_0000790 Is Involved in Pulmonary Vascular Remodeling in Mice with HPH via MicroRNA-374c-Mediated FOXC1

**DOI:** 10.1016/j.omtn.2019.12.027

**Published:** 2020-01-10

**Authors:** Lei Yang, Huan Liang, Xianguo Meng, Li Shen, Zhanjiang Guan, Bingchang Hei, Haitao Yu, Shanshan Qi

**Affiliations:** 1 ICU, The Third Affiliated Hospital of Qiqihar Medical University, Qiqihar 161002, P.R. China; 2 Glorious Community, The Third Affiliated Hospital of Qiqihar Medical University, Qiqihar 161002, P.R. China

**Keywords:** mmu_circ_0000790, microRNA-374c, FOXC1, pulmonary artery smooth muscle cells, hypoxic pulmonary hypertension, Notch pathway

## Abstract

Recently, the identification of several circular RNAs (circRNAs) as vital regulators of microRNAs (miRNAs) underlines the increasing complexity of non-coding RNA (ncRNA)-mediated regulatory networks. This study aimed to explore the effects of mmu_circ_0000790 on the biological behaviors of pulmonary artery smooth muscle cells (PASMCs) in hypoxic pulmonary hypertension (HPH). The HPH mouse model and hypoxia-induced PASMC model were initially established, and the expression of mmu_circ_0000790 in the pulmonary vascular tissues and hypoxic PASMCs was determined using quantitative reverse transcriptase polymerase chain reaction (qRT-PCR). A series of *in vitro* experiments such as dual-luciferase, RNA pull-down, and RNA-binding protein immunoprecipitation (RIP) assays were conducted to evaluate the interactions among mmu_circ_0000790, microRNA-374c (miR-374c), and forkhead transcription factor 1 (FOXC1). The potential physiological functions of mmu_circ_0000790, miR-374c, and FOXC1 in hypoxic PASMCs were investigated through gain- and loss-of function approaches. Upregulated mmu_circ_0000790 was found in both the HPH-pulmonary vascular tissues and hypoxic PASMCs. Additionally, mmu_circ_0000790 could competitively bind to miR-374c and consequently upregulate the target gene of miR-374c, FOXC1. It was also observed that mmu_circ_0000790 induced proliferation and inhibited apoptosis of hypoxic PASMCs, which further promoted the pulmonary vascular remodeling in mice with HPH. Therefore, we speculate that mmu_circ_0000790 may serve as a prospective target for the treatment of patients with HPH.

## Introduction

Pulmonary arterial hypertension (PAH) is a progressively worsening disorder characterized by an abnormally elevated blood pressure within the pulmonary arteries, accompanied by severe symptoms such as shortness of breath, chest pain, and fainting or syncope.^1^^,^^2^ Hypoxic pulmonary hypertension (HPH) is recognized as a condition featured with an elevation in pulmonary vascular tone in combination with the structural remodeling of peripheral pulmonary arteries.^3^ Reports have flagged associations between expanding genetic alterations with PAH, such as bone morphogenetic protein receptor 2 (BMPR2) and eukaryotic translation initiation factor 2α kinase 4 (EIF2AK4).^4^^,^^5^ Several pathways are implicated in the process of HPH; however the underlying mechanism has not been adequately investigated. The patients with HPH show pathological changes such as dysfunction of smooth muscle cells, fibroblasts, as well as pulmonary artery endothelial cells, and the dysfunction is linked to the progressive obliteration of pulmonary arteries.^6^ The therapies for PAH include prescriptions of endothelin receptor antagonists, prostaglandins, phosphodiesterase-5 inhibitors, and soluble guanylate cyclase stimulators.^7^ Despite the advances made in the currently available therapies for PAH, an important unmet medical need emerges, as PAH presents with high mortality.^8^ In recent years, the utilization of circular RNAs (circRNAs) has been reported as important biological molecules for a better understanding of the disease mechanisms and for identification of biomarkers for diagnosis and therapy of disease.^9^

circRNAs have been considered as a class of regulatory transcripts that are derivatives of protein-coding exons.^10^, ^11^, ^12^ Typically, several circRNAs play vital roles in diseases, particularly in different types of cancers, which, therefore, constitute novel potential targets for both the diagnosis and therapeutics for diseases.^13^^,^^14^ Previous evidence has illustrated that overexpressed circRNAs are predominantly enriched in the ribonucleotide biosynthetic process and purine ribonucleotide biosynthetic process.^15^ A recent study demonstrated the functionality of hsa_circ_0022342 and hsa_circ_0002062 as the key circRNAs for the development of chronic thromboembolic pulmonary hypertension (CTEPH) and that modulation of their targets might be an effective strategy for treating CTEPH.^15^ However, the vital functions of circRNAs have not been completely elucidated to date. The most extensively studied mechanisms of circRNAs illustrate their abilities to function as microRNA (miRNA) sponges.^16^ Moreover, miR-374a has been identified to serve as a prognostic biomarker for the survival of patients with non-small-cell lung cancer (NSCLC) at an early stage.^17^ miR-374 has been found to directly target forkhead box O1 (FOXO1), a member of the forkhead box (FOX) family and then stimulate cell proliferation and invasion in osteosarcoma.^18^ FOX proteins are a superfamily of conserved transcriptional regulators, with functionality applicable along a wide spectrum of biological processes.^19^ Furthermore, a prior study has suggested that the overexpression of FOXC1 activates the development of NSCLC, which may provide insight on a valuable marker for the prediction of the outcome of patients with this type of lung cancer.^20^ Based on this aforementioned literature, we hypothesized that the potential interactions among mmu_circ_0000790, miR-374c, and FOXC1 might influence the progression of HPH.

## Results

### Successful Establishment of HPH Mouse and Hypoxic Pulmonary Artery Smooth Muscle Cell (PASMC) Models

**Figure 1:**
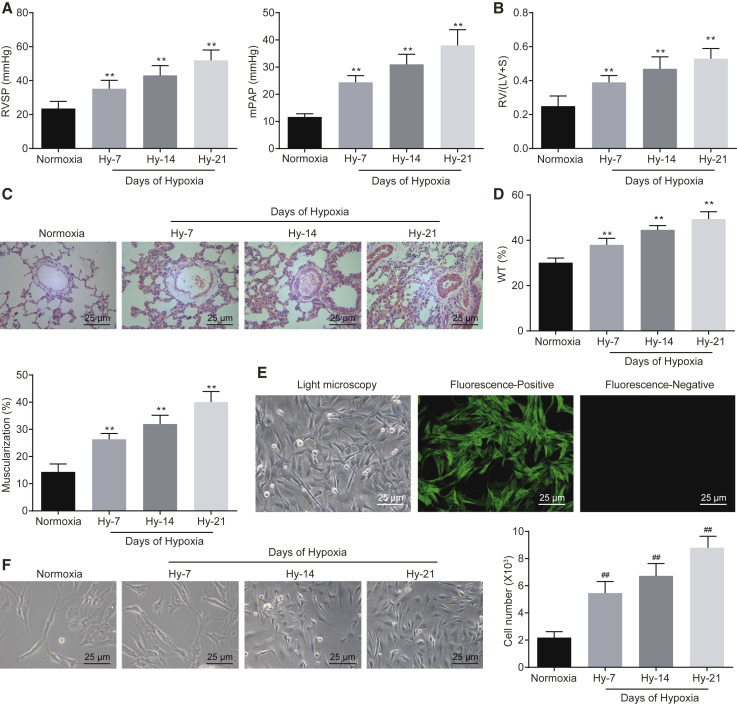
Successful Establishment of HPH Mouse and Hypoxic PASMC Models (A) RVSP and mPAP measured by a pressure sensor in normoxia and hypoxia groups. (B) RV/(LV + S) of heart in mice of normoxia and hypoxia groups. (C) Morphologic changes of small vessels in lung of normoxia and hypoxia mice by H&E staining (original magnification, ×400). (D) The wall thickness and muscularization of pulmonary arteries of mice in normoxia and hypoxia groups. (E) Morphologic characteristics of isolated PASMCs identified under a light microscope and expression of α-actin, a molecular marker of PASMCs, detected by immunofluorescence staining (original magnification, ×400); the PASMCs at passage 3 were “peak valley,” and the cells were stained by antigen and epitope. (F) Morphologic characteristics and the number of hypoxic PASMCs in each group (original magnification, ×400). **p < 0.01 versus the normoxia mice; ^##^p < 0.01 versus normoxia cells. N = 5. The data (mean ± standard deviation) among multiple groups were compared using one-way ANOVA. Each cell experiment was run in triplicate independently. HPH, hypoxic pulmonary hypertension; PASMCs, pulmonary artery smooth muscle cells; RVSP, right ventricular systolic pressure; mPAP, mean pulmonary arterial pressure; RV, right ventricle; LV, left ventricle; S, septum; H&E, hematoxylin and eosin; ANOVA, analysis of variance.

Right ventricular systolic pressure (RVSP) and mean pulmonary arterial pressure (mPAP) were measured using a pressure sensor in the normoxia and hypoxia groups. Successful establishment of HPH and hypoxic PASMC models was evaluated by determining the heart weight and the pulmonary vessels of the mice using hematoxylin and eosin (H&E) and immunofluorescence staining. The results of various hemodynamic indexes of mice with HPH showed that RVSP and mPAP in the hypoxia group were higher compared to the normoxia group, and the values of these indexes were elevated with an elevation in the continuous feeding days (p < 0.01; Figure 1A). Similarly, the ratio of right ventricle (RV) to left ventricle (LV) + septum weight (S) in the hypoxia group was notably higher than in the normoxia group (p < 0.01; Figure 1B), suggesting that the index value was elevated with an elevation in the continuous feeding days. The results of H&E staining showed that the wall thickness and muscularization of pulmonary arteries in the hypoxia group were higher than those observed in the normoxia group, and the lumen area was shrunken with an elevation in the continuous feeding days (p < 0.01; Figures 1C and 1D). Then, the PASMCs isolated from the pulmonary artery of normoxia mice were observed under a fluorescence microscope and identified using immunofluorescence staining. The results suggested that the PASMCs at passage 3 were in a “peak-valley” shape (p < 0.01; Figure 1E), which was in consistency with the fundamental morphological characteristics of PASMCs in mice. Additionally, the cytoplasm was reflective of green fluorescence, with the presence of a filamentous substance in the cytoplasm, i.e., actin. The purity of the isolated mouse PASMCs was quantified to be higher than 99%. Furthermore, the number of PASMCs was evidently elevated with an increased duration of hypoxia culture (Figure 1F). The aforementioned results were indicative of successful HPH mouse and hypoxic PASMC model establishment.

### High Expression of mmu_circ_0000790 Is Found in HPH Mouse and Hypoxic PASMC Models

**Figure 2:**
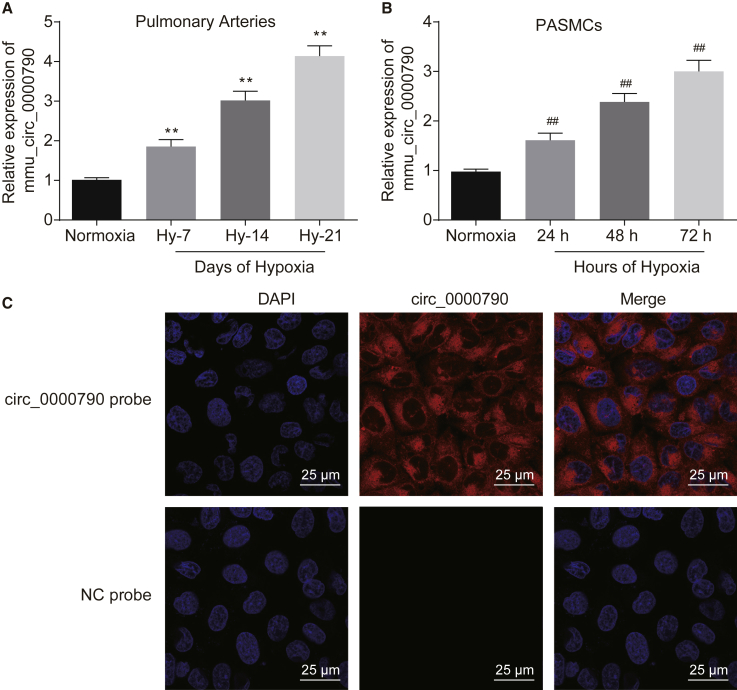
mmu_circ_0000790 Is Highly Expressed in Mice with HPH and Hypoxic PASMCs (A) Expression of mmu_circ_0000790 in pulmonary artery of normoxia and hypoxia mice detected by qRT-PCR. (B) Expression of mmu_circ_0000790 in hypoxic PASMCs examined by qRT-PCR. (C) The localization of mmu_circ_0000790 in hypoxic PASMCs examined by FISH (original magnification, ×400). **p < 0.01 versus the normoxia mice; ^##^p < 0.01 versus normoxia cells. N = 5. The data (mean ± standard deviation) among multiple groups were compared using one-way ANOVA. Each cell experiment was run in triplicate independently. HPH, hypoxic pulmonary hypertension; qRT-PCR, quantitative reverse transcriptase polymerase chain reaction; PASMC, pulmonary artery smooth muscle cell; FISH, fluorescence *in situ* hybridization; ANOVA, analysis of variance.

In the HPH and hypoxic PASMC models, the results of quantitative reverse transcriptase polymerase chain reaction (qRT-PCR) suggested that the expression of mmu_circ_0000790 increased over an increased duration of feeding/culture in the hypoxia group, and it was critically higher than in the normoxia group, which peaked at the highest level after hypobaric hypoxia for 21 days (Figure 2A) and hypoxia for 72 h (Figure 2B), respectively (p < 0.01). Therefore, we selected 21 days as the experimental cycle for the subsequent animal experimentation and 72 h in the cell culture. Meanwhile, fluorescence *in situ* hybridization (FISH) results indicated that mmu_circ_0000790 was localized in the cytoplasm of hypoxic PASMCs (p < 0.01, Figure 2C). These results suggested that mmu_circ_0000790 was extremely expressed in mice with HPH.

### Low Expression of mmu_circ_0000790 Inhibits Proliferation and Migration while Inducing Apoptosis of PASMCs from Mice with HPH

**Figure 3:**
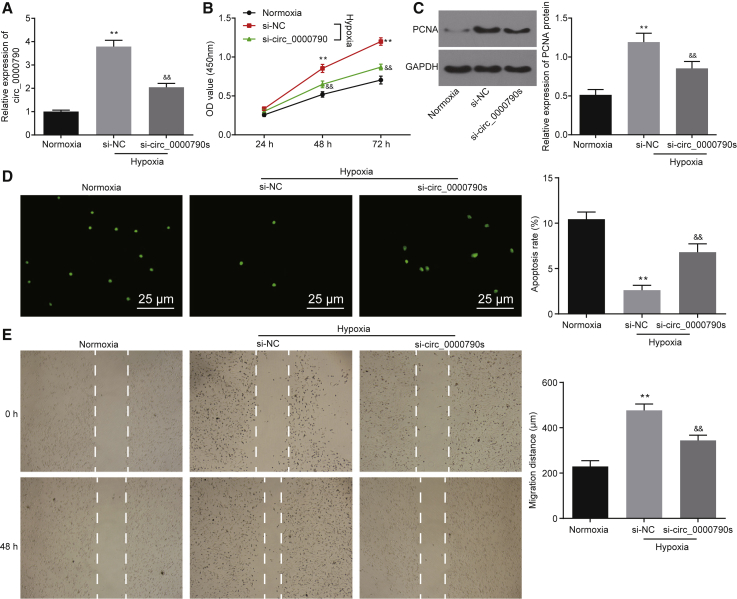
Silencing of mmu_circ_0000790 Suppresses Proliferation and Migration while Inducing Apoptosis of Hypoxic PASMCs (A) mmu_circ_0000790 expression in PASMCs of each group detected by qRT-PCR. (B) Proliferative ability of PASMCs in each group measured by a CCK-8 assay. (C) Protein expression of PCNA in PASMCs in each group detected by western blot analysis. (D) Apoptosis of PASMCs in each group measured by TUNEL assay (original magnification, ×400). (E) Migration ability of PASMCs detected by scratch test. **p < 0.01 versus the normoxia mice; ^##^p < 0.01 versus normoxia cells. N = 5. The data (mean ± standard deviation) among multiple groups were compared using one-way ANOVA, and those among groups at different time points were compared using ANOVA of repeated measurements. The experiment was run in triplicate independently. HPH, hypoxic pulmonary hypertension; PASMC, pulmonary artery smooth muscle cell; qRT-PCR, quantitative reverse transcriptase polymerase chain reaction; CCK-8, Cell Counting Kit-8; PCNA, proliferating cell nuclear antigen; TUNEL, terminal deoxynucleotidyltransferase-mediated dUTP nick end labeling; ANOVA, analysis of variance.

In assessment of the high expression of mmu_circ_0000790 in hypoxic PASMCs, we used the RNA silencing technique to knock down the expression of mmu_circ_0000790 to further investigate the molecular mechanism of mmu_circ_0000790 in hypoxic PASMCs. The results of qRT-PCR were assertive of successful silencing of mmu_circ_0000790 (p < 0.01, Figure 3A). According to the results of a Cell Counting Kit-8 (CCK-8) assay, western blot analysis, terminal deoxynucleotidyltransferase-mediated dUTP nick end labeling (TUNEL) assay, and scratch test, the cell viability, proliferation, and migration were elevated while the apoptosis rate of hypoxic PASMCs was lowered in the mmu_circ_0000790 and small interfering RNA (siRNA) negative control (si-NC) groups compared to the normoxia group (p < 0.01). In contrast to the si-NC group, the cell viability, proliferation, and migration were lowered while the apoptosis rate of hypoxic PASMCs was elevated in the mmu_circ_0000790 group (p < 0.01, Figures 3B–3E). In conclusion, silencing of mmu_circ_0000790 restrained proliferation and migration while also stimulating the apoptosis of hypoxic PASMCs.

### mmu_circ_0000790 Targets and Binds to miR-374c

**Figure 4:**
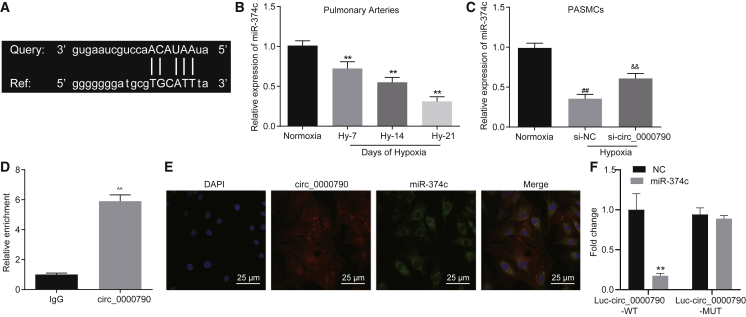
mmu_circ_0000790 Binds to miR-374c (A) Bioinformatics prediction of sequence containing the miR-374c binding site in mmu_circ_0000790 3′ UTR. (B) Expression of miR-374c in pulmonary artery of normoxia and hypoxia mice detected by qRT-PCR. (C) Expression of miR-374c in hypoxic PASMCs detected by qRT-PCR. (D) Enrichment of mmu_circ_0000790-specific probes detected by RIP assay. (E) Cellular localization of mmu_circ_0000790 detected by a FISH assay. (F) The binding of circ_0000790 to miR-374c confirmed by a dual-luciferase reporter gene assay. **p < 0.01 versus the normoxia mice; ^##^p < 0.01 versus normoxia cells; ^&&^p < 0.01 versus the si-NC; ˆˆp < 0.01 versus the IgG group. N = 5. The data (mean ± standard deviation) between two groups were compared using a t test, and those among multiple groups were compared with one-way ANOVA. Each cell experiment was run in triplicate independently. miR-374c, microRNA-374c; qRT-PCR, quantitative reverse transcriptase polymerase chain reaction; HPH, hypoxic pulmonary hypertension; PASMC, pulmonary artery smooth muscle cell; RIP, RNA-binding protein immunoprecipitation; FISH, fluorescent *in situ* hybridization; NC negative control; ANOVA, analysis of variance.

The bioinformatics analysis (starBase v2.0, CircInteractome) predicted the potential of mmu_circ_0000790 to bind to miR-374c in PASMCs. Therefore, a series of experiments was planned to ascertain that hypothesis. Figure 4A demonstrates the complementary sequence between mmu_circ_0000790 and miR-374c. Based on the results of qRT-PCR, in both HPH and hypoxic PASMC models, the expression of miR-374c was reduced (p < 0.01, Figure 4B), and its expression was lowered with an increase in hypoxic duration. In addition, the PASMCs treated with siRNA targeting mmu_circ_0000790 (si-circ_0000790) displayed an elevated expression profile of miR-374c in both HPH and hypoxic PASMC models (p < 0.01, Figure 4C). The results of an RNA-binding protein immunoprecipitation (RIP) assay with Argonaute2 (Ago2) protein as the antibody showed that the specific probe of mmu_circ_0000790 was enriched in combination with miR-374c and Ago2 protein (p < 0.01, Figure 4D). Meanwhile, FISH analysis revealed that mmu_circ_0000790 and miR-374c might be potentially co-localized in the cytoplasm (p < 0.01, Figure 4E). In addition, the results from a dual-luciferase reporter gene assay showed that miR-374c could inhibit the luciferase activity of circ_0000790 wild-type while circ_0000790 mutant (mut) remained unaffected (Figure 4F). These findings suggested the potential of mmu_circ_0000790 to bind to miR-374c.

### miR-374c Reverses the Promoting Role of mmu_circ_0000790 in the Development of Mice with HPH

**Figure 5:**
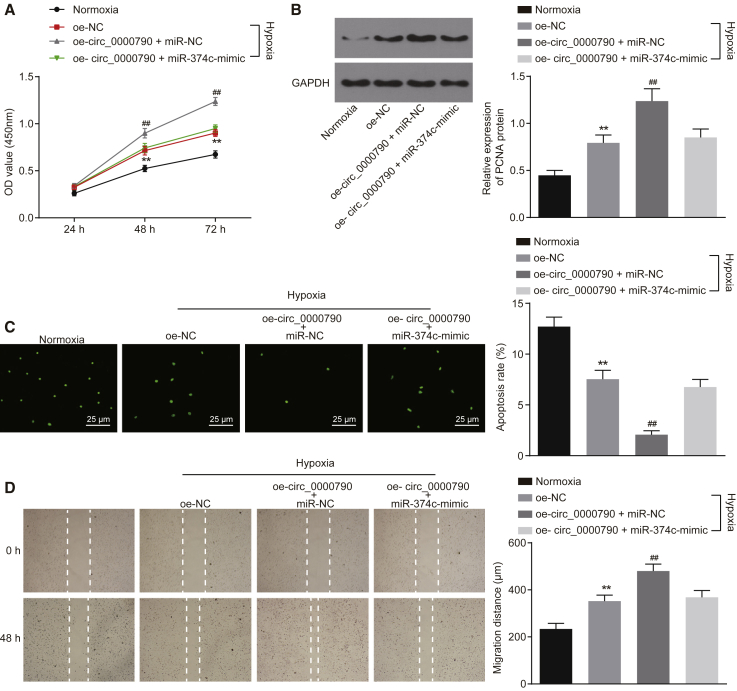
miR-374c Counteracts the Role of mmu_circ_0000790 in Promoting the Viability and Migration of Hypoxic PASMCs (A) Proliferative ability of PASMCs in each group by CCK-8 assay. (B) Expression of PCNA protein in PASMCs in each group by western blot analysis. (C) Apoptosis of PASMCs in each group by TUNEL assay (original magnification, ×400). (D) Migration ability of PASMCs determined by scratch test. **p < 0.01 versus the normoxia group; ^##^p < 0.01 versus the oe-NC and oe-circ_0000790 + miR-374c-mimic groups. The data (mean ± standard deviation) among multiple groups were compared with one-way ANOVA, and those among groups at different time points were compared by ANOVA of repeated measurements. The experiment was run in triplicate independently. miR-374c, microRNA-374c; HPH, hypoxic pulmonary hypertension; PASMC, pulmonary artery smooth muscle cell; CCK-8, Cell Counting Kit-8; PCNA, proliferating cell nuclear antigen; TUNEL, terminal deoxynucleotidyltransferase-mediated dUTP nick end labeling; MC, negative control; ANOVA, analysis of variance.

Further study focused on the involvement of miR-374c in respect to the functionality of mmu_circ_0000790 in hypoxic PASMCs. Based on the combination results of a CCK-8 assay, western blot analysis, TUNEL assay, and scratch test, the cell viability and migration ability were promoted while the apoptosis rate of hypoxic PASMCs was suppressed in the hypoxia group compared to the normoxia group (p < 0.01). On the contrary, the cell viability and migration ability were hindered while the apoptosis rate of hypoxic PASMCs was stimulated in the overexpressed mmu_circ_0000790 (oe-circ_0000790) + miR-374c-mimic group in contrast to the oe-circ_0000790 + miR-NC group (all p < 0.01, Figures 5A–5D). These results suggested that miR-374c counteracted the role of mmu_circ_0000790 along the stimulation of viability and migration of hypoxic PASMCs.

### mmu_circ_0000790 Regulates the Expression of FOXC1 by Competitively Binding with miR-374c, Thereby Affecting the Biological Characteristics of PASMCs in HPH Mice

**Figure 6:**
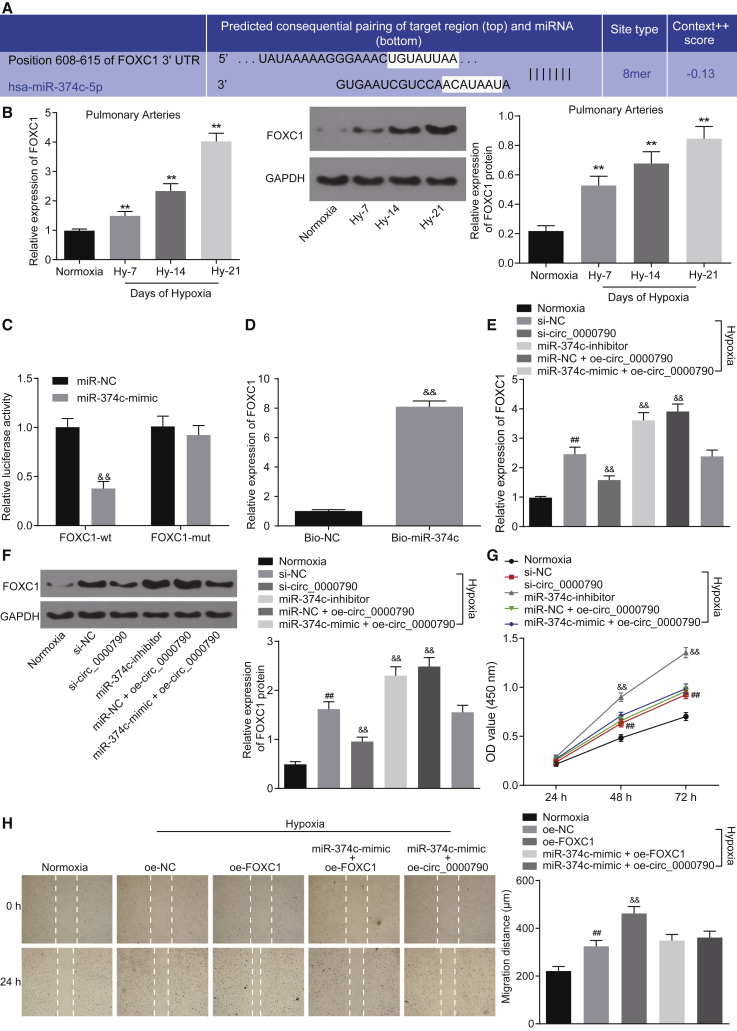
mmu_circ_0000790 Regulates FOXC1 Expression through Binding to miR-374c (A) Bioinformatics prediction of sequence complementarities between FOXC1 3′ UTR and miR-374c. (B) mRNA and protein expression of FOXC1 in mouse pulmonary artery detected by qRT-PCR and western blot analysis. (C) Validation of interaction of FOXC1 and miR-374c by dual-luciferase reporter gene assay. (D) Binding of FOXC1 and miR-374c detected by RNA pull-down assay. (E and F) mRNA and protein expression of FOXC1 in PASMCs detected by qRT-PCR and western blot analysis. (G) The proliferative ability of PASMCs detected by CCK-8 assay. (H) The migration ability of PASMCs detected by scratch test. **p < 0.01 versus the normoxia mice; ^##^p < 0.01 versus the normoxia cells; ^&&^p < 0.01 versus the miR-NC, bio-NC, si-NC, or oe-NC group. N = 5. The data (mean ± standard deviation) between two groups were compared using t test. Data among multiple groups were compared using one-way ANOVA, while those among groups at different time points were compared using ANOVA of repeated measurements. Each cell experiment was run in triplicate independently. FOXC1, forkhead box C1; miR-374c, microRNA-374c; UTR, untranslated region; qRT-PCR, quantitative reverse transcriptase polymerase chain reaction; PASMCs, pulmonary artery smooth muscle cells; CCK-8, Cell Counting Kit-8; NC, negative control; ANOVA, analysis of variance.

To further explore the mechanism of miR-374c in elimination of the promoting role of mmu_circ_0000790 in the development of HPH in mice, we conducted an array of experiments. *In silico* analysis revealed the presence of a binding site between miR-374c and FOXC1 in PASMCs (Figure 6A). Besides, qRT-PCR and western blot analysis showed that the mRNA and protein expression of FOXC1 in the pulmonary artery of HPH mice was notably higher than that in normoxia mice, and its expression increased over the duration of continuous feeding days (p < 0.01; Figure 6B). The results of a dual-luciferase reporter gene assay suggested that the luciferase activity of FOXC1-wild-type decreased in HEK293T cells transfected with the miR-374c mimic (p < 0.01; Figure 6C). Meanwhile, the results of an RNA pull-down assay suggested that compared with the bio-NC group, the enriched mRNA expression of FOXC1 was elevated in the bio-miR-374c group (p < 0.01; Figure 6D). In order to further investigate whether mmu_circ_0000790 regulates the expression of target gene FOXC1 by competing with miR-374c, qRT-PCR and western blot analysis were conducted for reference. The results showed that the mRNA and protein expression of FOXC1 in the hypoxic PASMCs was higher than that observed in the normoxia cells; the mRNA and protein expression of FOXC1 was increased in cells treated with miR-374c inhibitor or oe-circ_0000790 whereas it was decreased in cells treated with si-circ_0000790 (all p < 0.01; Figures 6E and 6F). The changes in the proliferation and migration ability of PASMCs after FOXC1 overexpression were determined by means of a CCK-8 assay and scratch test. The results indicated that the cell viability and migration ability of cells transfected with overexpressed (oe-)FOXC1 were increased under hypoxic conditions (p < 0.01; Figures 6G and 6H). These results suggested that FOXC1 could promote the proliferation and migration of PASMCs and it could be regulated by miR-374c and mmu_circ_0000790, respectively.

### mmu_circ_0000790 and miR-374c Participate in Pulmonary Vascular Remodeling in Mice with HPH through the Notch Pathway by Regulating FOXC1

**Figure 7:**
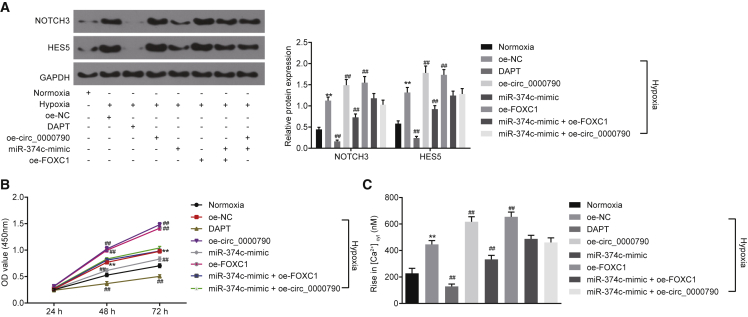
mmu_circ_0000790 and miR-374c Are Implicated in Pulmonary Vascular Remodeling in Mice with HPH by Regulating the Notch Pathway (A) Expression of Notch3 and HES5 in PASMCs detected by western blot analysis. (B) Proliferative ability of PASMCs as detected by a CCK-8 assay. (C) CCE peak value by measurement of Ca^2+^cyt. **p < 0.01 versus the normoxia mice; ^##^p < 0.01 versus the oe-NC group. N = 5. The data (mean ± standard deviation) among multiple groups were compared using one-way ANOVA, and those among groups at different time points were compared using ANOVA of repeated measurements. The experiment was run in triplicate independently. miR-374c, microRNA-374c; HPH, hypoxic pulmonary hypertension; PASMC, pulmonary artery smooth muscle cell; CCK-8, Cell Counting Kit-8; CCE, capacitative calcium entry; NC, negative control; ANOVA, analysis of variance.

Next, we explored the effects of mmu_circ_0000790 and miR-374c on the Notch pathway by western blot analysis, a CCK-8 assay, and measurement of cytosolic free Ca^2+^ concentration (Ca^2+^cyt). Western blot analysis illustrated that compared to the normoxia group, the expression of Notch3 and HES5 in the remaining groups was higher except for the DAPT group, the expression of Notch3 and HES5 in cells overexpressing circ_0000790 or FOXC1 was higher, while it was lowered in cells with overexpression of miR-374c and inhibition of the Notch pathway (all p < 0.01; Figure 7A). Similarly, the results of a CCK-8 assay revealed that relative to the normoxia group, the cell proliferation ability in the remaining groups was higher except for the DAPT group, the cell proliferation ability of cells with overexpression of circ_0000790 or FOXC1 was higher, while that with overexpression of miR-374c and inhibition of Notch pathway was lowered (all p < 0.01; Figure 7B). Furthermore, the results of measurement of Ca^2+^cyt suggested that the capacitative calcium entry (CCE) peak value was higher in the remaining groups, except for the DAPT group, in contrast to the normoxia group. The CCE peak value in cells with overexpression of circ_0000790 or FOXC1 was elevated, while that with overexpression of miR-374c and inhibition of Notch pathway was reduced (all p < 0.01; Figure 7C). In conclusion, both mmu_circ_0000790 and FOXC1 could promote the biological effects of an activated Notch pathway on hypoxic PASMCs that were inhibited by miR-374c.

### Low Expression of mmu_circ_0000790 or High Expression of miR-374c Retards the Development of HPH in Mice

**Figure 8:**
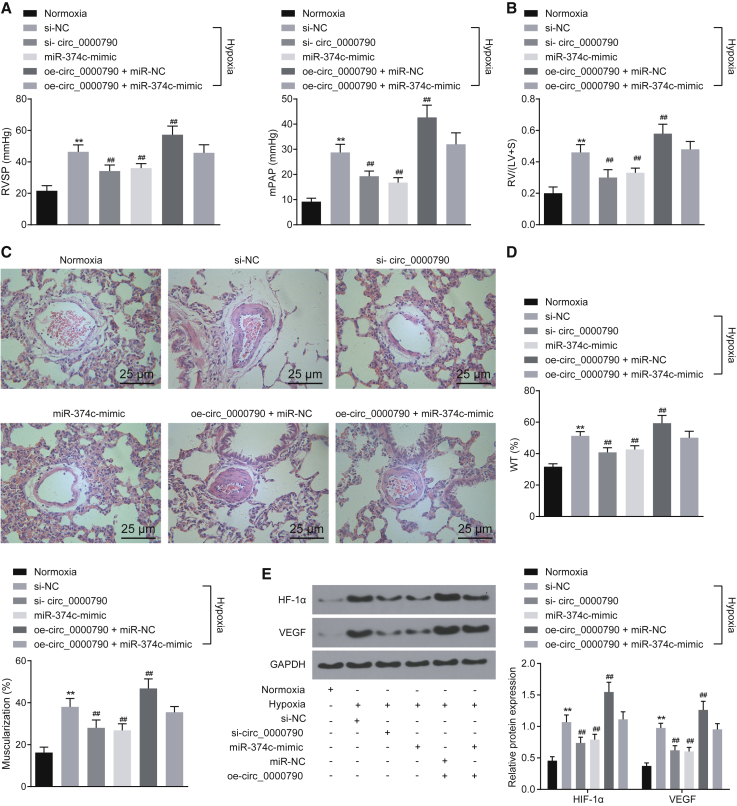
Either Low Expression of mmu_circ_0000790 or High Expression of miR-374c Disrupts the Development of HPH in Mice (A) RVSP and mPAP measured by a pressure sensor in normoxia and hypoxia groups. (B) RV/(LV + S) of heart in mice of normoxia and the hypoxia groups. (C) Morphologic changes of small vessels in lung of normoxia and hypoxia mice by H&E staining (original magnification, ×400). (D) Wall thickness and muscularization of pulmonary arteries of mice in normoxia and hypoxia groups. (E) Expression of HIF-1α and VEGF protein in pulmonary artery tissues in normoxia and hypoxia mice determined by western blot analysis. **p < 0.01 versus the normoxia mice; ^##^p < 0.01 versus the si-NC group. N = 5. The data (mean ± standard deviation) among multiple groups were compared using one-way ANOVA. miR-374c, microRNA-374c; HPH, hypoxic pulmonary hypertension; RVSP, right ventricular systolic pressure; mPAP, mean pulmonary arterial pressure; RV, right ventricle; LV, left ventricle; S, septum; H&E, hematoxylin and eosin; HIF-1α, hypoxia inducible factor-1α; VEGF, vascular endothelial growth factor; NC, negative control; ANOVA, analysis of variance.

The plasmids or synthetic RNAs including si-circ_0000790, miR-374c-mimic, oe-circ_0000790, si-NC, or miR-NC were injected into the mice via the tail vein using Entranster *in vivo* transfection reagents. The relative indexes of HPH were detected after continuous hypoxia/normoxia feeding conditions for 21 days. As shown in Figures 8A and 8B, compared with the normoxia group, the RVSP, the mPAP, and the ratio of right ventricle were increased in all hypoxia groups, which were also higher in the oe-circ_0000790 + miR-NC group relative to the si-NC group (all p < 0.01). Similarly, the results of H&E staining showed that the wall thickness and muscularization of pulmonary arteries were higher while the lumen area was lower in the hypoxia groups than in the normoxia group (all p < 0.01). The wall thickness and muscularization of pulmonary arteries were higher in the oe-circ_0000790 + miR-NC group compared to the si-NC group (all p < 0.01; Figures 8C and 8D). Then, the expression of hypoxia inducible factor-1α (HIF-1α) and vascular endothelial growth factor (VEGF) in pulmonary artery tissues of mice in each group was detected by means of western blot analysis. The results indicated that compared with the normoxia group, the expression of HIF-1α and VEGF was increased in all hypoxia groups, and the expression of HIF-1α and VEGF was also higher in the oe-circ_0000790 + miR-NC group in contrast to the si-NC group (all p < 0.01; Figure 8E). The data collected indicated that either downregulated mmu_circ_0000790 or upregulated miR-374c could prevent the development of HPH in mice.

## Discussion

**Figure 9:**
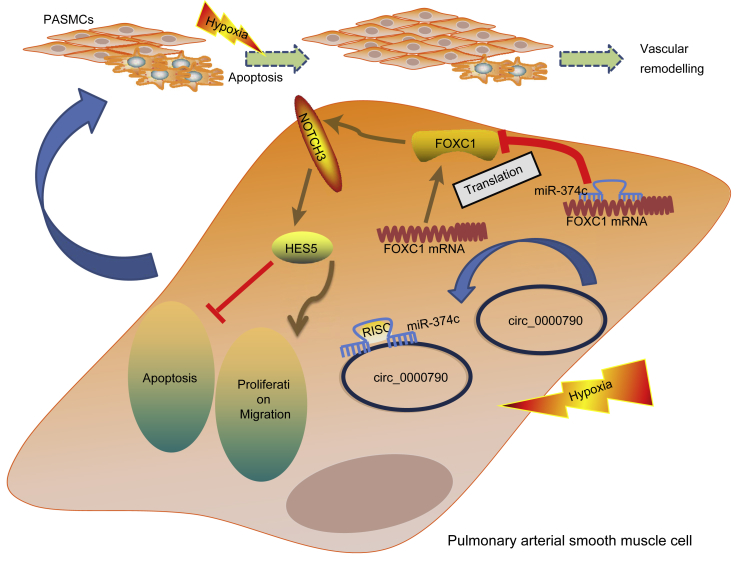
The mechanism investigation suggests that mmu_circ_0000790 regulates the expression of FOXC1 and the Notch pathway through binding to miR-374c, thus affecting the biological characteristics of hypoxic PASMCs, such as proliferation, apoptosis, and migration. FOXC1, forkhead box C1; miR-374c, microRNA-374c; HPH, hypoxic pulmonary hypertension; PASMCs, pulmonary artery smooth muscle cells.

Studies of HPH have increased recently, which results from more cases of polycythemia, pulmonary arterial remodeling, and hypoxic pulmonary vasoconstriction.^21^ Numerous studies have endeavored to elucidate the development of HPH, but only a few of them explain its pathogenesis distinctly. In order to better understand the molecular mechanism behind the pathogenesis of HPH, we designed this study with an aim of investigating the effects of mmu_circ_0000790 on the viability and migration of hypoxic PASMCs via miR-374c-mediated regulation of FOXC1. Collectively, our findings demonstrated that mmu_circ_0000790 regulated the expression of FOXC1 by binding to miR-374c, consequently affecting the cellular physiological processes of hypoxic PASMCs, such as proliferation, apoptosis, and migration (Figure 9).

One of the most significant findings of our study suggested the presence of an extensive expression of mmu_circ_0000790 in both pulmonary vascular tissues of HPH mice and hypoxic PASMCs, and suppression of mmu_circ_0000790 impeded the viability and migration while it simultaneously promoted the apoptosis of hypoxic PASMCs. Recently, accumulating evidence has flagged the involvement of upregulated circRNAs in the regulation of tumor progression.^22^ An existing study has suggested the presence of an elevated expression of circRNA_100876 in NSCLC tissues relative to their adjacent non-tumorous tissues.^23^ Elevated expression of hsa_circ_0013958 has been found in lung adenocarcinoma (LAC) tissues, cells, and plasma, and it can stimulate proliferation and invasion while simultaneously inhibiting the apoptosis in LAC cells.^24^ Additionally, various circRNAs are indicated for overexpression in non-cancer diseases, such as circRNA Cdr1 in myocardial infarction and Cdr1 as in diabetes.^25^^,^^26^

Also, our study revealed that mmu_circ_0000790 regulated the expression of FOXC1 by binding to miR-374c. Several circRNAs can function as sponges of miRNAs to regulate the expression of miRNA targets, thereby contributing to the competing endogenous RNA (ceRNA) network.^9^ Studies have flagged the potential of the circRNA-miRNA-mRNA axis as an emerging regulatory mechanism in human disease.^27^ In non-cancer diseases, heart disease and depressive disorders are associated with circRNAs. For instance, an activated axis of circ-HRCR-miR-223-ARC impedes hypertrophic responses.^28^ The axis of circRNA-CER/MMP13-miR-136 serves as a regulator of chondrocyte extracellular matrix (ECM) degradation.^29^ Furthermore, in cancer diseases, Zhu et al.^24^ have demonstrated the functionality of hsa_circ_0013958 as a ceRNA of miR-134 to upregulate oncogenic cyclin D1, which is of pivotal utility in the development of NSCLC. Furthermore, Huang et al.^30^ have proposed that miR-374c-5p exercised its functionality by targeting the FOXC1 3′ untranslated region (3′ UTR) and inhibiting FOXC1 expression, which underlines the vitality of miR-374c-5p in regulating cervical cancers by targeting FOXC1. Additionally, another functional representative circRNA, ciRS-7, is capable of binding to miR-7 to reduce its expression levels and thereby amplify the levels of miR-7-targeted transcripts.^31^ Therefore, circRNAs are significant participants along the progression of several diseases.

Moreover, the results from our study indicated that mmu_circ_0000790 and miR-374c are of important functionality in pulmonary vascular remodeling in mice with HPH by activation of the Notch pathway via FOXC1. The Notch pathway is an intrinsic pathway responsible for the regulation of communications among cells and controls cell differentiation in the embryonic and adult stages.^32^ As previously reported, inhibition of the Notch signaling using dibenzazepine, a γ-secretase inhibitor, is dose-dependently capable of reducing the proliferation and migration of human pulmonary arterial endothelial cells (hPAECs).^33^ Additionally, studies support that the interactions between FOXC1, the Notch signals, and VEGF control the expression of vascular genes and facilitate tumor angiogenesis in multiple steps.^34^ Furthermore, miR-374a demonstrates inverse functionality in NSCLC cells (SPCA-1 and H1975) by directly targeting PTEN to initiate the Wnt/β-catenin and Ras signaling in combination with its downstream cascade signals.^35^ Along the progression of one subtype of HPH, CTEPH, the target genes of hsa-miR-942-5p are predominantly enriched in cancer-related pathways, while the target genes of hsa-miR-940 are notably enriched in the ErbB signaling pathway, thereby signifying the importance of these pathways in CTEPH.^15^ Several analyses have identified associations among various other molecular pathways with hypoxia and vascular remodeling. The phosphatidylinositol 3-kinase (PI3K)-Akt signaling pathway plays a fundamental role in HPH by facilitating the proliferation of endothelial cells in compliance with smooth muscle cells.^36^^,^^37^ Meanwhile, the Hippo, Wnt, AMP-activated protein kinase (AMPK), as well as the HIF-1 signaling pathways have been demonstrated in associations with the development of HPH.^38^^,^^39^ These findings indicate the potential of circRNAs as key modulators along the molecular mechanism of HPH and that specific circRNAs could be contemplated as diagnostic and therapeutic targets of HPH in the clinical setting.

To conclude, we found the potential of mmu_circ_0000790 as a significant regulator in HPH development through its regulation on the miR-374c-FOXC1-Notch axis. The identification of a novel differentially expressed circRNA, mmu_circ_0000790, is a promising step for a better understanding of HPH progression. However, due to the differences in the sequence information of human and mouse circ_0000790 caused by the inconsistency of species, we mainly focused on the experiments and mechanisms in animal models. More studies in clinical settings are needed for further verification of our experimental results to determine whether the mmu_circ_0000790-mediated miR-374c-FOXC1 axis has a therapeutic effect on hypoxia-induced HPH.

## Materials and Methods

### Ethics Statement

This experiment was conducted with approval of the Animal Ethics Committee of The Third Affiliated Hospital of Qiqihar Medical University. All animal experiments in this study were in strict accordance with the protocols stated in the *Guide for the Care and Use of Laboratory Animals* published by the US National Institutes of Health. Appropriate measures were taken to minimize the number and suffering of animals.

### Establishment of HPH Mouse Model and Hypoxic PASMC Model

Several healthy male C57BL/6 mice (Shanghai Jiesijie Experimental Animal, Shanghai, China) aged 6–8 weeks and weighing 22–25 g were acquired for our experiment, with 8 mice in the normoxia group and 45 mice in the hypoxia group. Mice in the hypoxia group were housed in a hypobaric room for 7, 14, or 21 days, designated as the Hy-7, Hy-14, and Hy-21 groups, respectively. The intake valve was adjusted to maintain the pressure at 380 mmHg (0.5 atm) with 10% O_2_ and sufficient airflow passage through the chamber to prevent the accumulation of CO_2_ and NH_3_. Mice in the normoxia group were housed at normal pressure (760 mmHg) and normal oxygen at room temperature. All mice had free access to food and water, with homeostatic temperature at 25°C for 12-h alternating day/night cycles. Three weeks later, the mice were euthanized and the related indices were measured.

As for the establishment of hypoxic PASMC models, PASMCs from the hypoxia group were cultured at 37°C for 24, 48, or 72 h, respectively, under 3% O_2_, 5% CO_2_, and 92% N_2_. PASMCs from the normoxia group were cultured at 37°C under 21% O_2_, 5% CO_2_, and 74% N_2_, respectively.

### Measurement of Hemodynamics

Following PASMC model establishment, the mice were weighed and anesthetized using an intraperitoneal injection with 3% sodium pentobarbital. The mice were then fixed on an operating table appropriate for small animals, and the abdominal cavity was incised to expose the diaphragm. Then, a 2-mm syringe needle treated with heparin was inserted into the right ventricle through the diaphragm. The pressure transducer was connected and a physiological instrument was employed to detect the RVSP and mPAP. The experiment was repeated three times independently.

### H&E Staining

After euthanasia, the trachea of mice was separated. Next, the left lung lobe of mice was ligated and the right lung was fixed by conducting perfusion with 4% paraformaldehyde, followed by an additional regimen of fixation with 4% paraformaldehyde for 48 h. The samples were then embedded in paraffin, routinely sectioned, and subjected to H&E staining. The pathological changes in the pulmonary arterioles were observed under a light microscope. An IPPS 6.0 image processing system was adopted to detect the arterial wall thickness, after which the mean wall thickness (WT) of the pulmonary arteriole and the external diameter (ED) of the vessel were calculated, and the percentage of WT was calculated based on the formula: WT % = (2 × WT/ED) × 100%. The experiment was repeated three times independently.^40^

### *In Silico* Analysis

Differentially expressed circRNAs were screened from the pulmonary artery tissues of HPH mice by conducting circRNA chip screening. RNA22 (https://cm.jefferson.edu/rna22/Interactive/) was adopted to predict the binding sites of miR-374c in the mmu_circ_0000790 and 3′ UTR of FOXC1 mRNA. A bioinformatics database (http://starbase.sysu.edu.cn) was employed to retrieve any information on the interaction between circRNA and miRNA. In addition to this, the CircNet (http://circnet.mbc.nctu.edu.tw/) website was also employed to speculate the potential regulatory mechanism of mmu_circ_0000790.

### Isolation and Identification of PASMCs

The isolation of PASMCs was conducted in strict accordance with the protocols of existing reports.^41^ The coverslips were prepared with detection of the α-actin expression and the morphological characteristics of PASMCs by immunofluorescence staining. Five different visual fields were randomly selected for observation under a confocal fluorescence microscope, and the purity of PASMCs was calculated.

### RNA Isolation and Quantitation

Total RNA was extracted from tissues and cells using TRIzol reagent (10296010, Invitrogen, Carlsbad, CA, USA) according to the manufacturer’s instructions. The concentration, purity, and integrity of the extracted RNA were determined by Nano-Drop ND-1000 spectrophotometry and 1% agarose gel electrophoresis. The primers used in the study were synthesized by Beijing Genomics Institute (BGI) (Shenzhen, China) (Table 1). The miRNA-specific complementary primer was synthetized using the miRNA-specific reverse transcription (RT) primers from the TaqMan miRNA reverse transcription kit (4366596, Applied Biosystems, Foster City, CA, USA) and TaqMan miRNA assay quantitative PCR kit (4427975, Applied Biosystems, Foster City, CA, USA). The expression of miR-374c was measured by means of TaqMan miRNA assays according to the provided instructions. The expression of miR-374c was standardized using U6 RNA. The reverse transcription was performed in accordance with the provided instructions of the EasyScript First-Strand cDNA Synthesis SuperMix (AE301-02, TransGen Biotech, Beijing, China). Fluorescence quantitative PCR was conducted in accordance with the provided instructions of the SYBR Premix Ex Taq II kit (RR820A, Takara, Dalian, China). The qRT-PCR experiment was conducted using the ABI 7500 fluorescence quantitative PCR instrument (Applied Biosystems, Foster City, CA, USA). The expression of target genes was calculated based on the relative quantitative method (2^−ΔΔCt^).

**Table 1:** Primer Sequences for qRT-PCR

Gene	Primer Sequences (5′→3′)
miR-374c	F: GATAATACAACCTGCTAAGT
	R: ATAATACAACCTGCTAAGTG
U6	F: GCTTCGGCAGCACATATACT
	R: TTCACGAATTTGCGTGTCAT
mmu_circ_0000790	F: AGCGCCCGTGGGCATGTATTAG
	R: AAACCAACCCGGTGAGCTCCCT
FOXC1	F: GACGGAGAACGGTACGTGT
	R: GTCATAGACGAAAGCCCCCG
GAPDH	F: GTTGTCTCCTGCGACTTCA
	R: GCCCCTCCTGTTATTATGG

F, forward; R, reverse; qRT-PCR, quantitative reverse transcriptase polymerase chain reaction; miR-374c, microRNA-374c; GAPDH, glyceraldehyde phosphate dehydrogenase.

### Cell Treatment

Cells were treated with si-circ_0000790, oe-circ_0000790, miR-374c-mimic, oe-miR-374c, miR-374c-inhibitor, oe-FOXC1, and corresponding negative controls (si-NC, mimic-NC, inhibitor-NC, and oe-NC) respectively. The pSilencer 4.1-CMV neo vector or Pegfp-4.1N was applied as the backbone vectors, which were provided by Sangon Biotech (Shanghai, China). Additionally, the siRNA was obtained from the Sigma-Aldrich Mission shRNA (short hairpin RNA) library (St. Louis, MO, USA).

The PASMCs at passage 3 were cultured under hypoxic conditions for 12 h. Cells at a density of 1 × 10^6^ were treated with 50 μg of miR-374c plasmid or inhibitor or NC in 100 μL of Lipofectamine 2000 transfection reagent (11668019, Invitrogen, Carlsbad, CA, USA) based on the manufacturer’s instructions. Lipofectamine RNAiMAX transfection reagent (13778030, Invitrogen, Carlsbad, CA, USA) was adopted for siRNA transfection. Next, the transfected cells were positioned in a hypoxia chamber (air condition with 3% O_2_ and 5% CO_2_) for 72 h, and the PASMCs at passage 3 cultured under normal oxygen content were used as control.

Entranster *in vivo* animal transfection reagent (25 μL, 18668-11-1, Engreen, Beijing, China) was diluted by the addition of 25 μL of 10% glucose solution. The diluted transfection reagent was combined with the diluted nucleic acid solution and allowed to react for 15 min at room temperature. The nucleic acids (12.5 μg) were supplemented with 10% glucose solution (w/v; 25 μL) and then injected into the mice via the tail vein at one-third of the distal end.

Cells were cultured in SmGM-2 containing 5% fetal bovine serum (FBS) (S00725, Gibco-BRL/Invitrogen, Carlsbad, CA, USA). HEK293T cells were obtained from the American Type Culture Collection (ATCC) (https://www.atcc.org/) and cultured using Dulbecco’s modified Eagle’s medium (DMEM) containing a combination of 10% FBS (10100147, Gibco-BRL/Invitrogen, Carlsbad, CA, USA), with 100 U of penicillin/streptomycin (15140122, Gibco-BRL/Invitrogen, Carlsbad, CA, USA) added to the medium per milliliter. The cells were cultured in a hypoxia chamber for 48 h and then passaged. The cells at passage 3 were selected for subsequent experimentation.

### Immunofluorescence Staining

After the cell confluence reached approximately 30%–40%, the medium was replaced by SmGM-2 containing 5% FBS (S00725, Gibco-BRL/Invitrogen, Carlsbad, CA, USA). Next, the cells were dewaxed, rehydrated, washed, and blocked/permeabilized using phosphate-buffered saline (PBS). For detection of the proliferating cells, lung sections were subjected to overnight incubation at 4°C with the control immunoglobulin G (IgG) or with 200 μL of rabbit anti-mouse α-actin (Ab5694, Abcam, Cambridge, MA, USA), followed by addition of the secondary antibody donkey anti-rabbit DyLight 549 (SA5-10064, Invitrogen, Carlsbad, CA, USA) and nuclear fluorescent dye Yo-Pro-1 (Y3603, Invitrogen, Carlsbad, CA, USA). Finally, the lung sections were observed under a laser confocal microscope.

### FISH

The subcellular localization of mmu_circ_0000790 and the co-localization of mmu_circ_0000790 and miR-374c were determined using a FISH kit (BIS-P0001, Guangzhou Bersin Biotechnology, Guangzhou, China).^42^ The PASMCs were taken and marked. The slides were then baked at 50°C for 2–3 h, after which the samples on the slides were isolated, denatured by immersing in 2× sodium citrate buffer (standard saline citrate [SSC]) for 2–3 min, immediately dehydrated using ethanol (70%, 85%, and 95%) (3 min/time), and air-dried. Then, the mmu_circ_0000790 probe (5′-CCT CCC TCG AGT GGC CCA ACC AA-3′) hybridization solution labeled by digoxigenin was added to cover the cells on the slides. The NC probe was used as NC (5′-TGG GCA TGT ATT AGC TCT AGA-3′) (Invitrogen, Carlsbad, CA, USA). The slides were subsequently covered and the edge was sealed using rubber cement. After 5 min of denaturing in a water bath at 83°C, the slides were subjected to 42°C and hybridized for 16 h. Then, the rubber cement was removed from the slides, which was placed in 2× SSC and shaken until the slide dropped down. Next, the slides were immersed in 2× SSC three times (5 min/time), soaked in 70% ethanol for 3 min, and then dried naturally in conditions devoid of light. The slides were subjected to staining using 4′,6-diamidino-2-phenylindole (DAPI) for 5–10 min in conditions devoid of light, and then washed twice with ice-cold PBS. The fluorescence images were documented under a laser confocal scanning microscope. All images were captured under a Zeiss LSM 880 NLO (2 + 1 with BIG) confocal microscope system (Leica Microsystems, Mannheim, Germany).^42^

### CCK-8 Assay

Proliferation of the PASMCs was measured by a CCK-8 assay (CK04, Dojindo, Kunamoto, Japan) using the CCK-8 kit. The cells were seeded in 96-well plates with 2,000 cells/100 μL/well in SmGM-2 medium containing 5% FBS. The CCK-8 reagents (10 μL) were added to each well, and then the optical density (OD) value of each well at the wavelength of 450 nm was measured with a microplate reader.

### TUNEL Assay

About 5 × 10^7^ cells/mL were fixed using a 4% paraformaldehyde solution at room temperature for 30 min, to which PBS containing 0.3% Triton X-100 was further added. TUNEL detection solution was prepared based on the provided instructions of the TUNEL cell apoptosis detection kit (C1088, Beyotime Biotechnology, Shanghai, China). The samples were then observed under a fluorescence microscope at the excitation wavelength ranging from 450 to 500 nm, and the emission wavelength for detection was from 515 to 565 nm. The experiment was repeated three times independently.

### Scratch Test

PASMCs were seeded into six-well plates at a density of 2.5 × 10^4^ cells/cm^2^. After 24 h of incubation, the cell growth was arrested overnight by addition of the starvation medium (SmGM-2 medium containing 5% FBS). Then, the images at 0 and 48 h were documented under microscopic observation. The width of each scratch was measured using ImageJ software, and the migration ability of cells in each group was evaluated by comparing the scratch width of each group.^43^

### Western Blot Analysis

The protein concentration of each protein sample was determined using the bicinchoninic acid (BCA) protein assay kit (P0012S, Beyotime Biotechnology, Shanghai, China). Total cellular lysate was resolved by conducting sodium dodecyl sulfate polyacrylamide gel electrophoresis (SDS-PAGE) (P0015, Beyotime Biotechnology, Shanghai, China), and it was then transferred onto a polyvinylidene difluoride (PVDF) membrane (FFP36, Beyotime Biotechnology, Shanghai, China). A membrane blockade was conducted using 5% bovine serum albumin (BSA) for 2 h at 37°C. Then, the membrane was incubated with the corresponding primary/secondary antibodies (rabbit anti-glyceraldehyde 3-phosphate dehydrogenase [GAPDH] polyclonal antibody [G9545, 0.2 g/mL], rabbit anti-Notch3/FOXC1 polyclonal antibody [N5038/SAB4500920, 1:1,000], rabbit anti-HIF-1α polyclonal antibody [SAB2702132, 1:10,000], rabbit anti- proliferating cell nuclear antigen (PCNA)/VEGF/HES5 polyclonal antibody [AV03018/SAB1306008/H9288] [Sigma-Aldrich, Steinheim, Germany], and rabbit secondary antibody [ab6721, 1:20,000, Abcam, Cambridge, UK]). Lastly, the membrane was developed using a development kit (P0020, Beyotime Biotechnology, Shanghai, China). The images were analyzed using ImageJ. With reference to GAPDH as the internal control, the relative protein expression was measured.

### RIP Assay

As previously described,^44^ the binding of mmu_circ_0000790 and miR-374c to Ago2 was determined using a RIP kit (17-701, Millipore, Milford, MA, USA). After preparation of antibody-coated protein A, PASMCs were treated with 200 μL of RIP lysis buffer and co-precipitated with 900 μL of RIP assay buffer in antibody-coated magnetic beads. Finally, RNA was purified by the conventional TRIzol process and then determined by qRT-PCR.

### RNA Pull-Down Assay

The combination of miR-374c and FOXC1 was determined using a Pierce magnetic RNA-protein pull-down kit (20164, Pierce, Milwaukee, WI, USA). After routine detachment, centrifugation, and precipitation of PASMCs, the cells were lysed with RIP lysis buffer. According to the provided instructions of the kit, miR-374c, miR-NC, FOXC1-wild-type, and FOXC1-mut were labeled with biotin, then enriched with magnetic beads labeled with streptavidin and subjected to incubation with lysate overnight at 4°C. At last, the RNA was purified based on the conventional TRIzol process, and then the RNA content was determined by qRT-PCR.

### Dual-Luciferase Reporter Gene Assay

The relationship between circ_0000790 and miR-374c, miR-374c, and FOXC1 was analyzed using a biological prediction website (https://cm.jefferson.edu/rna22/Interactive/), and the dual-luciferase reporter gene assay was employed to further verify whether circ_0000790 or FOXC1 was the target gene of miR-374c. According to the predicted binding site, the artificially synthesized mRNA 3′ UTR fragments of circ_0000790 and FOXC1 were inserted into plasmid pGL3-basic (P2129, Shanghai Hewu Biotechnology, Shanghai, China). The complementary sequence mutation site of the seed sequence was designed on FOXC1-wild-type and constructed into the reporter plasmids, which were comprised of a combination of Luc-circ_0000790-wild-type, Luc-circ_0000790-mut, FOXC1-wild-type, and FOXC1-mut, respectively. The correctly sequenced luciferase reporter plasmids were co-transfected into HEK293T cells with miR-374c and NC, respectively. The luciferase activity was measured using the dual-luciferase reporter gene assay kit (E1910, Promega, Madison, WI, USA). Fluorescence intensity was measured using a GloMax 20/20 luminometer fluorescence detector (E5311, Shaanxi Zhongmei Biotechnology, Shanxi, China). The experiment was repeated three times independently.

### Measurement of Ca^2+^cyt

Cells were loaded with fura-2-acetoxymethyl ester (fura 2-AM) (B6984, Guangzhou Yongjin Biotechnology, Guangzhou, China) and then superfused using the standard bath solution. The fluorescence signals were continuously monitored using an Intracellular Imaging fluorescence microscopy system and then documented on a computer for later analysis. Ca^2+^cyt was calculated from the fura-2 fluorescence emission at excitation wavelengths of 340 and 380 nm (F340/F380) using a method.^45^

### Statistical Analysis

The SPSS 21.0 statistical analysis software (IBM, Armonk, NY, USA) was employed for data analysis. The measurement data were presented as mean ± standard deviation. All experiments were performed in triplicate independently. Comparison between two groups was analyzed using the t test, while comparison among multiple groups was processed using the one-way analysis of variance (ANOVA). Comparison among multiple groups of data at different time points was analyzed using ANOVA of repeated measurements. A value of p < 0.05 was considered to be statistically significant, while a value of p < 0.01 was considered to be extremely statistically significant.

## Author Contributions

L.Y., H.L., and X.M. designed the study. L.S., Z.G., and B.H. collated the data, carried out data analyses, and produced the initial draft of the manuscript. L.Y., H.L., B.H., H.Y., and S.Q. contributed to drafting the manuscript. All authors have read and approved the final submitted manuscript

## Conflicts of Interest

The authors declare no competing interests.
